# CT Imaging in Predicting Ovarian Torsion: Report of Two Cases, With and Without Infarction

**DOI:** 10.7759/cureus.17082

**Published:** 2021-08-11

**Authors:** Sweta Singh, Prakash K Sasmal, Krishnan Nagarajan

**Affiliations:** 1 Obstetrics and Gyenacology, All India Institute of Medical Sciences, Bhubaneswar, IND; 2 General Surgery, All India Institute of Medical Sciences, Bhubaneswar, IND; 3 Radiodiagnosis, Jawaharlal Institute of Postgraduate Medical Education & Research, Puducherry, IND

**Keywords:** ovarian torsion, adnexal torsion, computed tomography, ovarian infarction, twisted pedicle

## Abstract

Ovarian torsion is one of the common abdominal and gynecologic surgical emergencies with serious morbidity in the form of ovarian infarct or necrosis. Various imaging modalities like USG, CT, and MRI have been used in the evaluation of ovarian torsion. Two middle-aged females presented with lower abdominal pain. Imaging showed an ovarian cystic lesion in the first patient and thickened and twisted ovarian pedicle or ‘helical swirling’ sign in the second. Intraoperatively, the first case turned out to be a 180* twist with mild ovarian edema and the second showed more than 720* torsion with ovarian infarction. The CT findings of twisted pedicle with pericystic fat stranding might be predictive of hemorrhagic infarction in cases of torsion.

## Introduction

The ovary is a female pelvic organ suspended in the pelvic peritoneal cavity by three peritoneal folds or ligaments - the infundibulopelvic fold or suspensory ligament, which attaches the ovary to the lateral pelvic wall, the utero-ovarian ligament connecting the ovaries to the uterus, and the mesovarium connecting the ovaries to the broad ligament of the uterus [1]. Torsion of an ovary, ovary with a tube, or rarely the tube alone can occur at any age. It is considered more common in children due to normal ligamentous laxity [2]. Ovarian torsion can occur with normal ovaries, ovaries having functional cysts, or with space-occupying lesions in the ovary such as dermoids (commonly) or other cystic or solid neoplasms [3, 4]. Torsion may impede the venous outflow causing edema and congestion. Later, the arterial inflow may be compromised ending in hemorrhagic infarction or necrosis. Imaging findings may vary depending on whether the torsion is partial or total, intermittent or progressive, and collateral supply of ovary from other vessels like the uterine artery branches [1].

## Case presentation

Case 1

**Figure 1:**
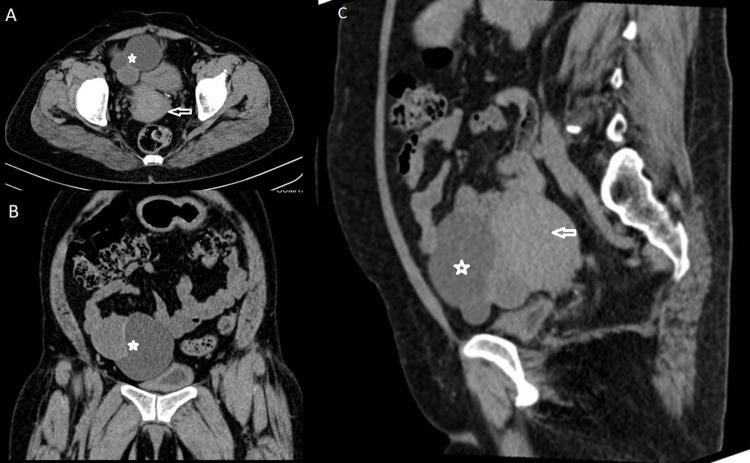
Case 1 CT transverse section (A), coronal section (B), and sagittal section (C) reformations showing multiloculated cystic lesion (marked with asterisks) anterolateral to the uterus (arrows in A & C) on right side

A 48-year-old female presented with right lower abdominal pain for one month. She gave a history of a right adnexal cyst of small size. On USG, a multiloculated cystic lesion was noted in the right adnexa, which was relatively tender and had a mildly swollen appearance. The lesion showed normal surrounding vascularity with part of the normal ovary in continuity with the lesion. A CT scan confirmed the findings and did not show any surrounding fat stranding or fluid collection (Figure 1).

**Figure 2:**
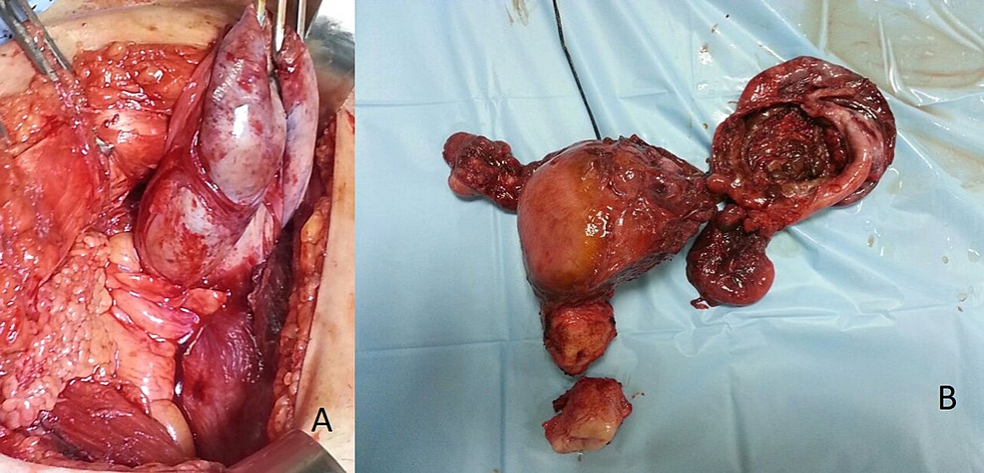
Case 1 (A) Intra-operative photograph and (B) gross specimen, showing partial twist and edematous cystic lesion (cut surface open in B) removed along with the uterus

No para-uterine or adnexal soft tissue thickening area was noted. On surgical exploration, a 180* twist of the cystic lesion and the ovary was seen (Figure 2). The ovary was grossly normal with only mild enlargement.

Case 2

**Figure 3:**
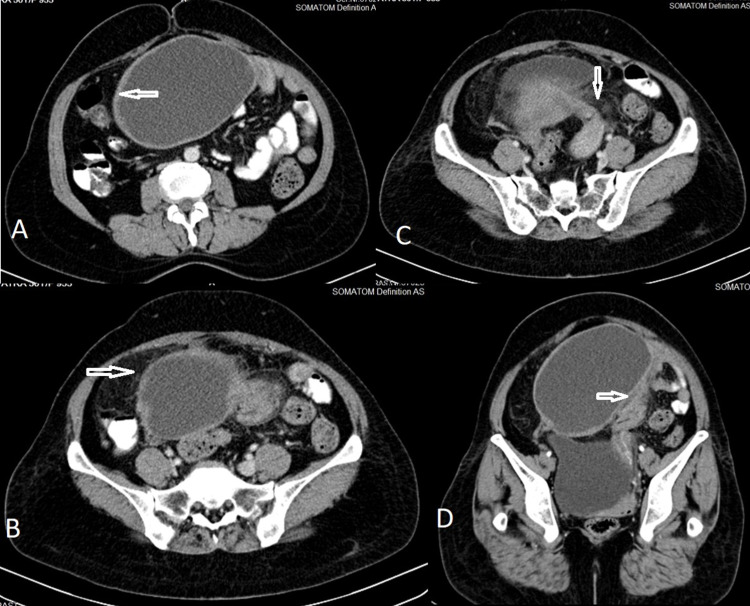
Case 2 CT axial sections (A, B, C) and oblique coronal reformation (D) showing a unilocular cystic lesion, anterosuperior to the uterus with thick walls (arrow in A), perilesional fat stranding (arrow in B), and twisted pedicle with ‘helical swirl’ appearance in continuous with left cornu of the uterus (arrows in C & D)

**Figure 4:**
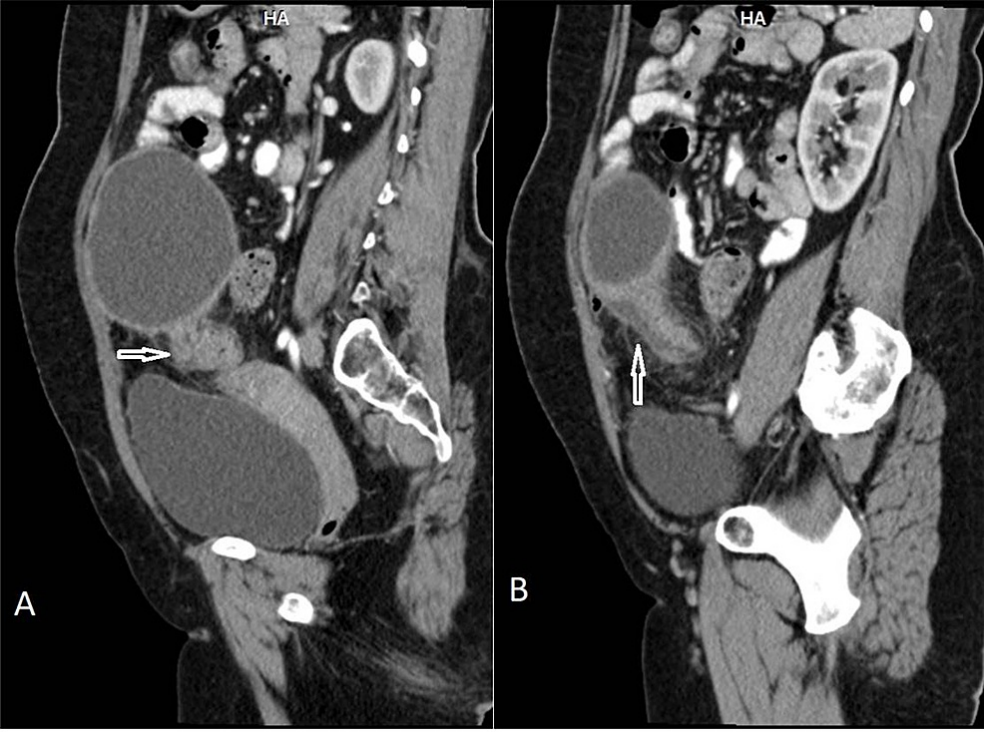
Case 2 CT sagittal reformations (A & B) showing ‘helical swirl’ appearance of twisted pedicle between the cystic lesion and the uterus (marked by arrows)

A 50-year-old female presented with a history of long-standing right lower abdominal discomfort that had increased since four days. She had moderate-to-severe right para-umbilical and lower abdominal pain with guarding. Ultrasonography revealed a large unilocular cystic lesion extending up into the para-umbilical region. The lesion showed increased peripheral vascularity and the right ovary was not separately visualized. The uterus and left ovary were normal. The cyst showed thick walls and subtle pericystic fat stranding, which are unusual for otherwise benign-looking features. There was helical soft tissue thickening that showed contrast enhancement extending from the lateral aspect of the uterus towards the cyst (Figure 3 and Figure 4).

**Figure 5:**
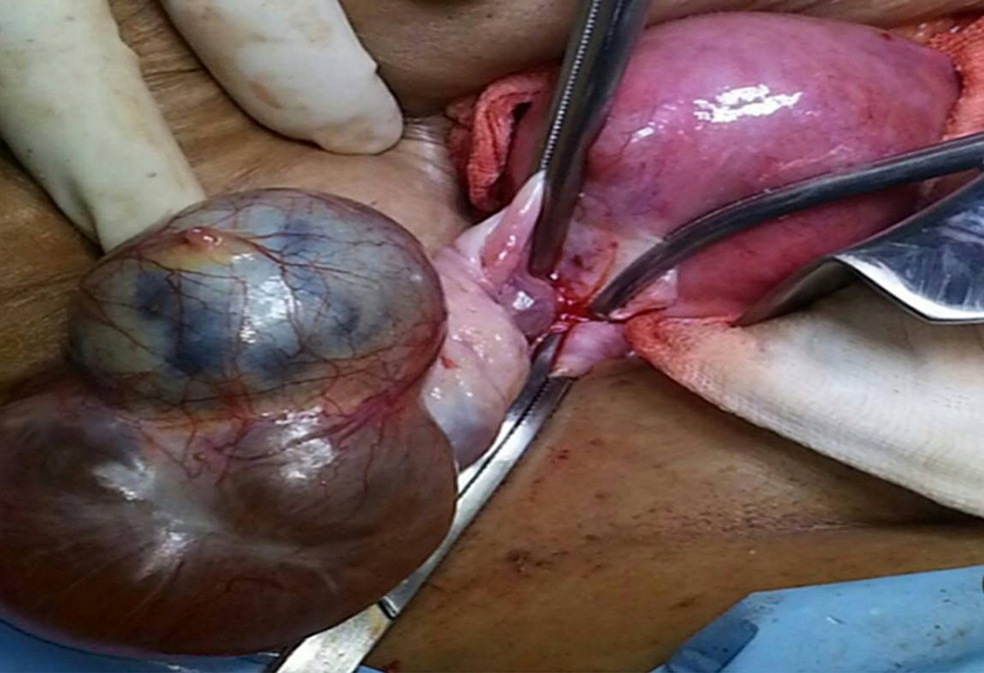
Case 2 Intra-operative photograph showing right ovarian cystic lesion with hemorrhagic infarction

**Figure 6:**
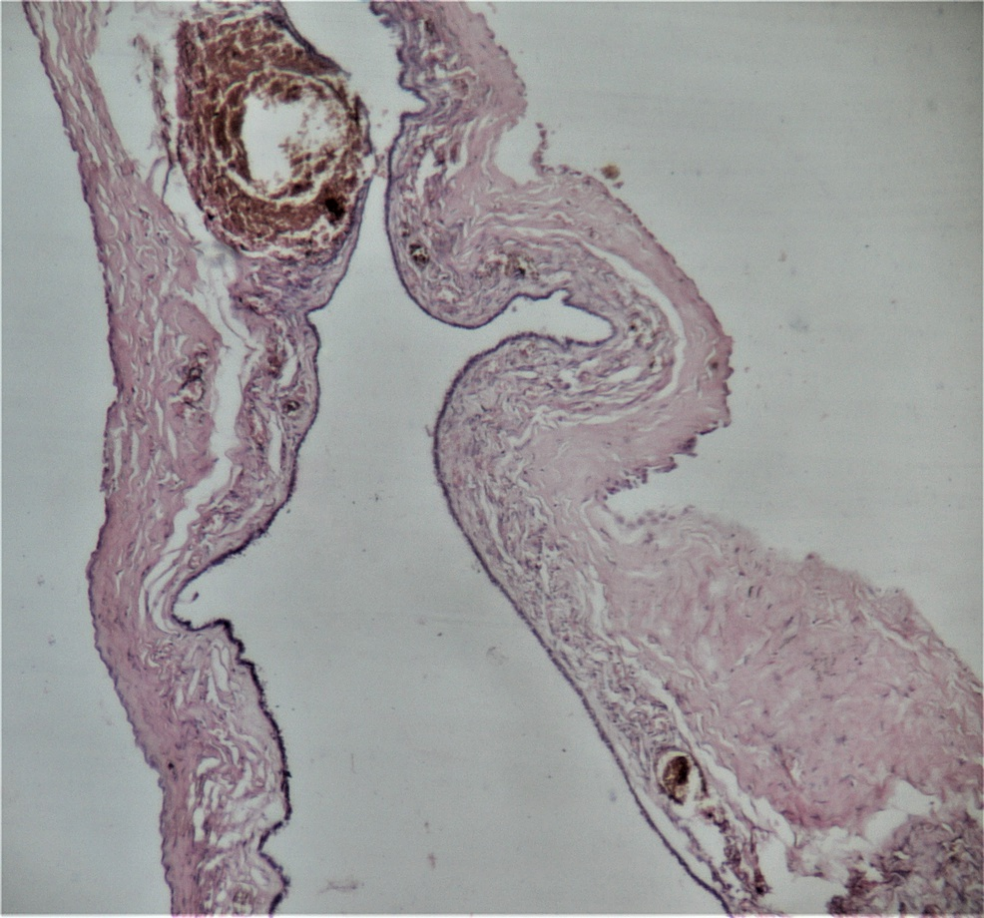
Case 2 Histopathological section (H & E stain; 4x magnification) showing ovarian cyst wall lined by ciliated cuboidal epithelium with focal denudation and perivascular hemorrhage around the congested blood vessel

There was a strong suspicion of torsion and the patient was taken for surgery. On surgery, the cyst showed more than two twists around its pedicle (nearly 720*) and it also showed hemorrhagic congestion (Figure 5). The histopathology was that of cystadenoma with infarct changes (Figure 6).

## Discussion

Ovarian torsion is one of the acute gynecologic surgical emergencies that need early identification for proper management [5]. The first choice of evaluation may be a USG, but it is limited by operator dependence. The USG findings are abnormal location of the ovary, either anterior to or above the uterus or in the pouch of Douglas, ovarian edema, enlargement, ovarian cyst/mass, abnormal flow, and free fluid [6]. Other less common findings are tubal enlargement and hemorrhagic foci within the ovary. Another study mentioned the sonographic ‘whirlpool' sign as a reliable sign of ovarian torsion [7]. However, the highest diagnostic accuracy achieved was 74.6 % due to many factors, the primary being operator-dependence and the question of the need for further workup when the sonography is negative [6].

CT and MRI have been used in the evaluation of torsion in more subacute settings. As early as 1994, Kimura et al. [8] described CT and MRI findings of uterine deviation, engorged vessels, minimal free fluid, and obliteration of fat in cases of torsion. They also mentioned hemorrhagic cases (ovarian infarct), converging vessels from the ovary to the uterus, draped vessels around the lesion, areas of hemorrhage, and non-enhancement. Intravascular gas within an ovarian mass is a very specific, but extremely rare, finding of CT, and only one report has been described till now [9].

CT is routinely used as a problem-solving modality in acute abdomen that can also help to rule out torsion [10]. Rhe et al. [11] also studied CT and MRI findings and found that adnexal/tubal thickening and thickened smooth wall of the cystic lesion were seen in those who underwent hemorrhagic infarction. Duigenan et al. [12], in their study of CT/MRI findings with pathologic correlation, found that the twisted pedicle sign or helical swirling sign was pathognomonic and most specific of torsion, though it is slightly difficult to identify. A recent study by Mondoul et al. [13] compared the diagnostic performance of CT signs and found that inter-utero-ovarian mass was an independent, accurate, and reliable finding of torsion. The awareness of this specific sign may help both clinicians and radiologists to specifically look for this in cases of acute abdomen with lower abdominal pain. Swenson et al. [14] compared USG and CT in emergency settings and mentioned that when CT findings of torsion are present, another imaging modality like USG is not only time-consuming but also can delay the treatment. Both our cases were on the right side and torsion is reported more on the right side. The reason ascribed to this is colonic position on the left pelvis and hypermobility of the cecum/distal ileum on the right side [11].

Ito et al. [15] first described that the torsion angle determines the incidence of necrosis in torsion. They retrospectively studied CT findings of torsion and divided the patients based on the angle of torsion into less than 360* and more than 360*. The twisted pedicle with tube and mesovarium between the uterus and ovary was described as ‘mass-like swelling’. They found that the twisted pedicle or mass-like swelling was seen mainly in those with torsion of more than 360* and developed ovarian necrosis. This necrosis due to hemorrhagic infarction also had, in few cases, an area of high density due to hemorrhage and absent enhancement. In our first case, CT showed a multiloculated cyst on the right side of the uterus without any other changes. The second case showed a right-sided unilocular cystic lesion pushed above the right cornu of the uterus and showed thickened walls, the helical swirling sign of a twisted pedicle, and minimal free fluid. Surgically these correlated with less than 180* twist in the first case and two rounds of twisting (720*) in the second. The first cyst showed only mild edematous swelling and the second lesion had changes of hemorrhagic infarction. It is to be seen by comparison of a larger number of patients with CT imaging whether this can be a reliable sign separating ovarian torsion with and without infarction or necrosis. Both of our patients were middle-aged and torsion is slightly less common at this age than in younger women. The reason is not known but it needs to be ascertained whether age-related ligamentous laxity or weakness could be a predisposing factor to torsion similar to its role in pelvic organ prolapse.

## Conclusions

CT findings of twisted pedicle with pericystic fat stranding may be useful to predict those cases of adnexal torsion leading to infarction by indicating more than 360* twisting of adnexal torsion and hence correlate with operative and gross findings of infarction in cases of torsion.
